# Comparing clinical responses and the biomarkers of BDNF and cytokines between subthreshold bipolar disorder and bipolar II disorder

**DOI:** 10.1038/srep27431

**Published:** 2016-06-07

**Authors:** Tzu-Yun Wang, Sheng-Yu Lee, Shiou-Lan Chen, Yun-Hsuan Chang, Liang-Jen Wang, Po See Chen, Shih-Heng Chen, Chun-Hsien Chu, San-Yuan Huang, Nian-Sheng Tzeng, Chia-Ling Li, Yi-Lun Chung, Tsai-Hsin Hsieh, I Hui Lee, Kao Chin Chen, Yen Kuang Yang, Jau-Shyong Hong, Ru-Band Lu

**Affiliations:** 1 Department of Psychiatry, National Cheng Kung University Hospital, College of Medicine, National Cheng Kung University, Tainan, Taiwan; 2 Department of Psychiatry, Kaohsiung Veterans General Hospital, Kaohsiung, Taiwan; 3 Graduate Institute of Medicine, College of Medicine, Kaohsiung Medical University (KMU), Lipid Science and Aging Research Center, KMU, Kaohsiung, Taiwan; 4 Institute of Allied Health Sciences, College of Medicine, National Cheng Kung University, Tainan, Taiwan; 5 Deprtment of Psychology, Asia University, Taichung, Taiwan; 6 Department of Child and Adolescent Psychiatry, Kaohsiung Chang Gung Memorial Hospital and Chang Gung University College of Medicine, Kaohsiung, Taiwan; 7 Addiction Research Center, National Cheng Kung University, Tainan, Taiwan; 8 Neurobiology Laboratory, NIH/NIEHS, Research Triangle Park, NC, USA; 9 Institute of Molecular Medicine, College of Medicine, National Cheng Kung University, Tainan, Taiwan; 10 Department of Psychiatry, Tri-Service General Hospital, National Defense Medical Center, Taipei, Taiwan; 11 Student Counseling Center, National Defense Medical Center, Taipei, Taiwan; 12 Institute of Basic Medical Sciences, National Cheng Kung University, Tainan, Taiwan; 13 Institute of Behavioral Medicine, College of Medicine, National Cheng Kung University, Tainan, Taiwan; 14 Department of Psychiatry, National Cheng Kung University Hospital, Dou-Liou Branch, Yunlin, Taiwan; 15 Center for Neuropsychiatric Research, National Health Research Institutes, Miaoli, Taiwan

## Abstract

Patients with subthreshold hypomania (SBP; subthreshold bipolar disorder) were indistinguishable from those with bipolar disorder (BP)-II on clinical bipolar validators, but their analyses lacked biological and pharmacological treatment data. Because inflammation and neuroprogression underlies BP, we hypothesized that cytokines and brain-derived neurotrophic factor (BDNF) are biomarkers for BP. We enrolled 41 drug-naïve patients with SBP and 48 with BP-II undergoing 12 weeks of pharmacological treatment (valproic acid, fluoxetine, risperidone, lorazepam). The Hamilton Depression Rating Scale (HDRS) and Young Mania Rating Scale (YMRS) were used to evaluate clinical responses at baseline and at weeks 0, 1, 2, 4, 8, and 12. Inflammatory cytokines (tumour necrosis factor [TNF]-α, transforming growth factor [TGF]-β1, interleukin [IL]-6, IL-8 and IL-1β) and BDNF levels were also measured. Mixed models repeated measurement was used to examine the therapeutic effect and changes in BDNF and cytokine levels between the groups. HDRS and YMRS scores significantly (P < 0.001) declined in both groups, the SBP group had significantly lower levels of BDNF (P = 0.005) and TGF-β1 (P = 0.02). Patients with SBP and BP-II respond similarly to treatment, but SBP patients may have different neuroinflammation marker expression.

Bipolar disorder (BP) is a severe mental illness and associated with significant morbidity and mortality^1^,^2^. The lifetime prevalence of BP in epidemiological surveys using the Diagnostic and Statistical Manual of Mental Disorders, Fourth Edition (DSM-IV) criteria is between 0.3% and 1.5% for bipolar I disorder (BP-I) and between 1.0% and 2.0% for bipolar II disorder (BP-II)^3^. However, growing evidence supports the existence of a spectrum of BP, which is far wider than recognized by current diagnostic nosology^4^. Although DSM-5 criteria require a minimum duration of 4 days of hypomania, a growing body of evidence suggests that the DSM criteria for BP-II on the requisite duration of hypomania symptoms are too strict because they exclude a substantial percentage of patients with varying manifestations of bipolar syndrome^4^,^5^,^6^,^7^,^8^,^9^. These patients who have a shorter hypomania are now classified as having “other specified bipolar and related disorder”(SBP; subthreshold bipolar disorder) in the DSM-5, with a high prevalence from 33.5% to 55% in the community^6^,^10^.

A wealth of epidemiological data provide important clinical information about the prevalence, severity, functional impairment, comorbidities, and characteristics of SBP and support its similarities with BP-II^4^,^5^,^6^,^11^,^12^,^13^,^14^. The most recognized evidence is from family studies, some of which have reported that a family history of bipolarity in patients displaying a shorter duration of hypomania is more in line with BP-II than with unipolar depression^6^,^14^,^15^. Neuropsychological tests also provide some evidence that patients with SBP performed equally to patients with BP-II in all of the cognitive domains^16^. In the BRIDGE study on a sample of 5635 patients with major depressive episodes^17^, the validity of short-duration hypomania (defined by 1–3 day episodes) was examined; the study found that patients with only 1-day hypomanic episodes still differed markedly from those with unipolar depressive disorder in clinical manifestation including age of onset, illness progression, resistance to treatment, and history of suicide attempts. Their finding confirmed the existence of short-duration hypomania which has a similar validity to longer hypomanic episodes^17^. Angst *et al*.^17^ suggested that the DSM criteria be revised to include short-duration (1–3 days) hypomanic episodes to allow much earlier recognition and treatment of BP. However, the BRIDGE study examines only clinical manifestations but not biological differences, and it has no follow-up design for examining different treatment response between patients with SBP and BP-II. Because of the limited information, uncertainties about optimal interventions, including pharmacotherapy, for SBP remain^9^. Therefore, investigating the clinical response and its possible pathophysiological changes in SBP is crucial.

BP is at least in part a neuroprogressive disorder in clinical observations^18^. The neuroinflammatory process interact with disturbances related to oxidative stress^19^ and neurotrophic mechanisms^20^ have been implicated in the neurobiological background and neuroprogressive processes of BP^18^. Activation of microglia has been supposed to play a major role in neuroinflammatory pathways^21^. A recent PET imaging study found increased microglial activity and neuroinflammation in the hippocampus area in BP subjects^22^. Upon activation, microglia can produce neurotoxic effects through production of proinflammatory cytokines^23^. In addition to elevated levels of cerebrospinal fluid (CSF) proinflammatory cytokine^24^, alterations in the peripheral levels of cytokines—tumor necrosis factor-α (TNF-α)^25^,^26^, transforming growth factor -β1 (TGF-β1)^27^, interleukin-6 (IL-6)^25^,^28^, interleukin-8 (IL-8)^29^ and interleukin -1β (IL-1β)^25^,^30^—which were found during mood episodes or euthymia in patients with BP. Thus, peripheral cytokine levels have been served as biomarkers for disease state and disease trait for BP^31^. However, the cytokine level is sensitive to multiple confounders^32^ and certain psychoactive medication, including mood stabilizers^33^,^34^,^35^, antidepressants^36^ and antipsychotics^37^, may affect the patient’s inflammatory status. Well-controlled prospective studies that do not use multiple combined medications, and that ensure a longitudinal follow-up the cytokine-level changes in the same patients, are required.

Moreover, neurotrophins, such as brain-derived neurotrophic factor (BDNF), are critical for neuronal survival and proliferation^18^. Post-mortem brains from patients with BP have significant lower than expected levels of BDNF, which might contribute to brain atrophy and progressive cognitive changes^38^. The reduction of serum BDNF levels is correlated with decreased BDNF levels in the brain^39^, further support that part of neurodegeneration in BP is related to a decrease in BDNF levels in acute episodes^40^ and a cumulative effects when the disorder progress^41^. The levels of BDNF appear to be normal in the early stages of the disorder in euthymic subjects, and decrease in the latter stages^42^, so serum BDNF displays as a biomarkers for both state markers during acute episodes and disease progression markers that accompany with clinical course^18^,^43^. Differences in the levels of inflammatory cytokines and BDNF in SBP and BP-II patients were never examined in the past. Following-up with SBP and BP-II patients undergoing standard treatment should allow us to clarify the potential of using inflammatory cytokines and BDNF levels as biomarkers to trace both the disease status and pharmacological response to therapeutic intervention.

In the prospective and longitudinal study, we investigated whether the levels of the potential disease biomarkers of cytokines and BDNF and the response to pharmacological interventions are different between patients with SBP and BP-II. We believe our findings will serve as initial biological evidence for the classification of SBP, which might help clinicians diagnose and treat patients with SBP.

## Methods

### Participants

The research protocol was approved by the Institutional Review Board for the Protection of Human Subjects at Tri-Service General Hospital and at National Cheng Kung University Hospital. The study was done in accordance with the ethical standards laid down in the 1964 Declaration of Helsinki. The procedures were fully explained to each participant before they were asked to sign the informed consent.

This study is a subgroup analysis of clinical trials for BP (Trial registration: NCT01188148 and NCT01188265 at https://register.clinicaltrials.gov/). The original study was a randomized, double-blind, controlled 12-week trial that investigated whether treating BP with valproic acid (VPA) plus add-on memantine or dextromethorphan is more effective than treating it with VPA alone^44^,^45^,^46^. The original study included BP-II patients with the 2-day cutoff for hypomanic episodes. Although some researchers^6^,^17^ have proposed that hypomania lasting for only 1 day might be sufficient for diagnosing hypomania, most of the studies^5^,^9^,^47^,^48^ suggested the 2-day cutoff for hypomanic episodes. We therefore selected the 2-day threshold because it has more support. Because the aim of the current study was to compare the pharmacological response and changes in cytokine and BDNF levels in shorter (SBP) and longer hypomania (BP-II) patients during the 12-week interventions, we used only patients from the placebo group for this subgroup analysis to avoid the influence of add-on memantine or dextromethorphan, which are not routinely used medications for treating BP. Only outpatients were recruited. All patients were initially evaluated by an attending psychiatrist and then underwent a more detailed interview by a clinical psychologist using the structured interview of the Chinese Version of the Modified Schedule of Affective Disorder and Schizophrenia- Life Time (SADS-L)^49^, which has good inter-rater reliability^50^, to determine the patient’s DSM-IV diagnosis. Patients with BP-II or with major depressive episodes and a history of subthreshold hypomania (more than 2-days but less than 4-days duration) were included. Patients with other major or minor mental illnesses, except BP-II or SBP, were excluded. We then subdivided the patients into SBP and BP-II groups for further analysis. The duration of hypomania was determined by “the most common duration” question based on a suggestion in a previous study^14^. Participants who could not recall the duration of hypomania were excluded from the current analysis. The number of BP-II patients in the placebo groups enrolled in these two trials was 171, but 82 of them had not answered the most common duration questions.

After the baseline assessment, the patients were given open-label VPA (500 mg and 1000 mg daily [50–100 μg/mL in plasma]) for one week. They were then randomly assigned to an add-on trial medication group or to the placebo group while continuing their open-label VPA treatment. Only placebo group patients were recruited for this study. The treatment period from the baseline to week 0 was lasted for one week. The plasma VPA level was checked after week 2. Limited use of risperidone (~6 mg/day), fluoxetine (10–20 mg/day), and the benzodiazepine (lorazepam; ~8 mg/day) was allowed as concomitant medication. We prescribed risperidone for patients with symptoms of hypomania, and lorazepam for patients with symptoms of anxiety or insomnia. The doses were adjusted according to each patient’s clinical manifestations, response, and tolerance. In cases of side-effects, intolerance, or clinical worsening, the patient was withdrawn from the study. Twenty of the participants also participated in group psychotherapy sessions during the follow-up. The severity of mood symptoms was assessed using the Young Mania Rating Scale (YMRS)^51^ and the Hamilton Depression Rating Scale (HDRS)^52^,^53^. The clinical treatment response was evaluated by research psychiatrists who had been trained in and were experienced in using the rating scales. Symptom severity was measured at baseline, and at weeks 0, 1, 2, 4, 8, and 12. After the pharmacological treatment had been initiated, plasma BDNF and cytokine levels were measured at baseline and at each visit when symptom severity was assessed.

### Measuring plasma cytokine levels

Twenty milliliters of blood was drawn from each participant. Plasma was isolated from the whole blood after it had been centrifuged at 3000 g for 15 min at 4 °C, and then it was immediately stored at −80 °C. Cytokine levels were quantified using an antibody pair assay system (Flexia; BioSource Intl., Camarillo, CA). Sample processing and data analysis were done according to the manufacturer’s instructions. The immunological parameters, TNF-α, TGF-β1, IL-6, IL-8, IL-1β, and BDNF levels were measured.

### Statistics

Pearson χ 2 analysis was used to examine the sex differences and other categorical variables. Fisher’s exact test was substituted for the χ 2 test when values were smaller than expected (<5). We used Student’s t tests to compare the differences of mean age, disease duration, and cytokines and BDNF levels between the SBP and BP-II groups at baseline and at the endpoint. YMRS and HDRS total scores were used as measures of response in each group. Potential prognostic factors included the different group effects, treatment duration (weeks 0–12), baseline YMRS and HDRS scores, disease duration, concomitant non-pharmacological therapy (group psychotherapy) and medications, sex, and age. Because there were repeated assessments, mixed models repeated measurement (MMRM) was used to control for time effects, psychopathology, and other related variables. Significance was set at P < 0.05. The changes from baseline HDRS and YMRS scores were analyzed using MMRM with autoregressive type of covariance and fixed effects for sex, age, visit, concomitant group psychotherapy and medications, and different hypomania duration subgroups. The changes of cytokines and BDNF levels in the SBP and BP-II groups were analyzed in the same way and controlled for symptom severity, time effects (treatment period from baseline to week 12), disease duration, concomitant group psychotherapy and medications, sex, and age. Differences from baseline were assessed based on the MMRM results. SPSS 18.0 was used for statistical analysis.

### Ethical approval

The research protocol was approved by the Institutional Review Board for the Protection of Human Subjects at National Cheng Kung University Hospital and Tri-Service General Hospital.

## Results

Of the 89 patients in the study, 48 had been diagnosed with BP-II and 41 with SBP. Thirty-seven patients could not complete the 12 weeks of treatment and withdrew from the study. The demographic data, e.g., sex and mean age, at baseline were not significantly different between the two groups (Table 1). Nor were disease duration and the severity of mood symptoms, based on HDRS and YMRS scores, significantly different (Table1). Plasma VPA levels, concomitant medication doses, and the rates of concurrent group psychotherapy are shown in Table 1. The rates of specific group psychotherapy and concomitant medication doses were not significantly different between the two groups (Table 1). At the beginning of the study, TNF-α, TGF-β1, and IL-8 levels were not significantly different. Baseline plasma cytokine levels of IL-1β were significantly higher in the SBP group (p = 0.03, Table1), but baseline IL-6 and BDNF levels were significantly lower in the SBP group (P = 0.02 and 0.03, respectively, Table 1). After 12 weeks of treatment, plasma cytokine levels were not significantly different between groups, but BDNF levels were still significantly lower in the SBP group (P = 0.02)(Table 1).

**Figure 1:**
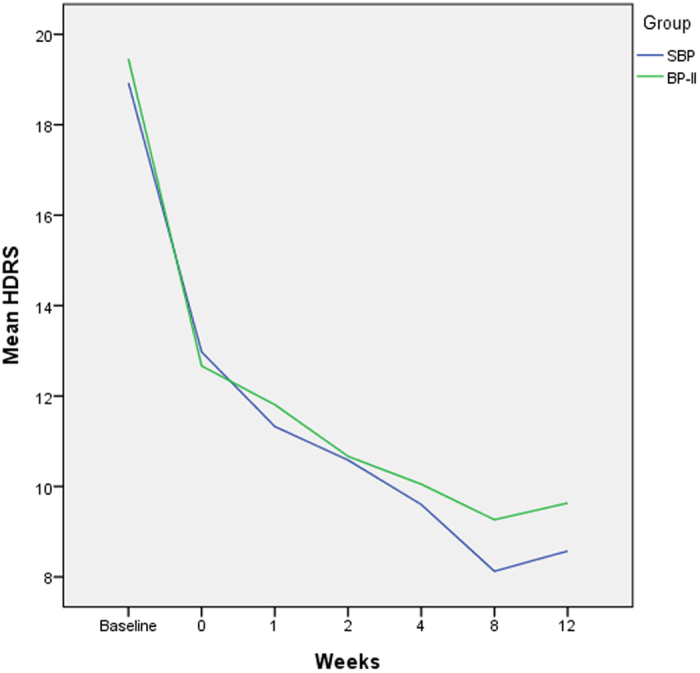
Improvement of the mean HDRS scores in the SBP and BP-II group during 12 weeks of pharmacological treatment.

**Figure 2:**
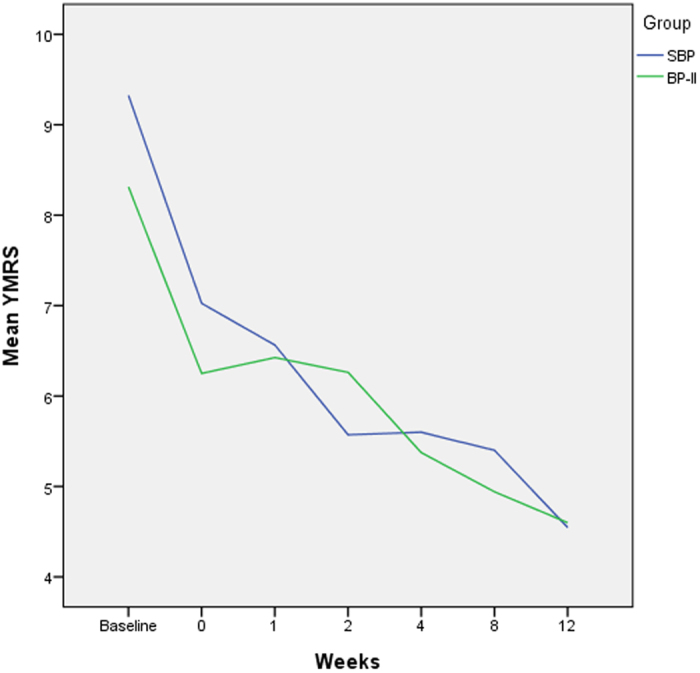
Improvement of the mean YMRS scores in the SBP and BP-II group during 12 weeks of pharmacological treatment.

We used MMRM to analyze the treatment effects in both group. HDRS and YMRS scores were significantly lower (P < 0.001, t = −11.43 and −7.24) after 12 weeks of pharmacological treatment (Figs 1 and 2). The changes in HDRS and YMRS scores were not significantly different between two groups, however (Table 2).

We also used MMRM to analyze the laboratory data. The analysis was controlled for covarying age, sex, treatment duration, disease duration, concomitant group psychotherapy and medications, and HDRS and YMRS scores. The BDNF level was a significantly lower in the SBP group than in the BP-II group during 12 weeks of treatment (P = 0.005). Changes in TGF-β1 levels were significantly different in the SBP group than in the BP-II group during the12 weeks of treatment (P = 0.02) (Table 2). Changes in the levels of other inflammatory cytokines were not significantly different after multiple covarying factors had been controlled for (Table 2).

## Discussion

To our knowledge, this is the first longitudinal study to compare the biomarkers of cytokine and BDNF levels and of SBP and BP-II patients’ treatment responses to mood stabilizers. We found that although the treatment responses to pharmacological therapy were equal between two groups, the BDNF and TGF-β1 biomarkers showed significant different level changes in both groups.

We found that the BDNF levels were significantly lower in the SBP group than in the BP-II group during the entire course of treatment, even after controlling for the possible effects of treatment, disease duration, and severity of mood symptoms. The BDNF levels were lower during acute mood episodes and recovered during euthymia in the early stage of BP, but in the late stage, BDNF levels were lower even during euthymic phase^18^,^43^,^54^. It is hypothesized that a lower BDNF level is associated with disease progress and the severity of mood symptoms in BP^18^. Although BDNF levels were significantly negatively correlated with YMRS scores and borderline negatively correlated with HDRS scores in the present study, this correlation became nonsignificant in MMRM after multiple confounders had been adjusted for. We hypothesized that a larger sample would confirm the association proposed in Beck *et al*.^18^ and tested the hypothesis that the disease severity and prognosis of SBP patients are not as mild as previously reported when compared with those of patients with longer-duration hypomania in BP-II^4^,^8^.

Studies have reported a proinflammatory imbalance with higher levels of the inflammatory markers in levels of IL-6^42^, TNF-α,^42^,^55^ and IL-1β^24^ at late stages of BP. Although baseline IL-1β and IL-6 levels were significantly different between the SBP and BP-II groups, the changes in IL-1β and IL-6 levels during the 12 weeks of treatment were not significantly different after controlling for the effects of concomitant medications and group psychotherapy. Additional studies to control these possible confounders with a larger sample size are needed to explore whether the difference becomes more significant.

Moreover, TGF-β1 is reported to be both an important modulator and suppressor of inflammatory cytokines^56^. Patients with BP have lower levels of TGF-β1 during acute episodes but increased levels after treatment^27^. A high initial plasma level of TGF-β1 also associated with a better prognosis during combination treatment with quetiapine and lithium in manic patients^57^. Our SBP patients had a persistently lower level of TGF-β1 during treatment (Table 2), which might indicate that an underlying proinflammatory imbalance is more difficult to improve in them than in BP-II patients, even with the same HDRS and YMRS clinical response scores.

Proinflammatory cytokines have also been reported to be negative regulators of hippocampal neurogenesis^20^. The crude data showed a positive correlation between BDNF and TGF-β1 and IL-6 in the current study. The association between the regulatory cytokine TGF-β1 and BDNF partly supports the hypothesis of a relationship between neuroinflammation and neurodegeneration in BP spectrum disorder, but findings for IL-6 do not. Although our data are comparable with Patas *et al*.^58^, who reported a significant association between IL-6 and BDNF levels in patients with major depressive disorder, some debates remained for lack of controlling disease states or clinical courses. Additional investigations on the topic of BDNF and cytokine interaction in a well-controlled mood episode might provide additional support for this hypothesis.

Why there were differences in neuroinflammation between SBP and BP-II patients in the present study is unknown. However, unadjusted analysis showed a trend of negative correlation between YMRS scores and BDNF and TGF-β1 levels. Although the correlation became nonsignificant after the analysis had been adjusted for multiple confounders, we hypothesized that the difference in BDNF and TGF-β1 between the two groups might be attributable to a mild, though nonsignificantly, higher YMRS scores in SBP patients. In addition, different disease courses, different prior treatments, disease relapse times, and severities might have affected the neuroinflammation markers^35^,^42^. We did not control for the smoking, BMI, or other metabolic parameters in current study, either. All of these factors might have affected our findings^58^. Because our objective is novel, our methods and findings are not comparable with other studies. Replication and confirmation of our results will be required.

Our study provides initial evidence that the treatment effect of pharmacological therapy for mood symptoms is not significantly different between SBP and BP-II patients. Our finding complements the BRIDGE Study by Angst *et al*.^17^ , which showed that patients with shorter and longer durations of hypomania did not significantly differ in their responses to treatment. Therefore, both studies support a broader definition of hypomania. Because a high rate of subthreshold hypomania in MDD patients has been reported^6^,^10^, our data also suggest a possibility of using mood stabilizers to treat patients with SBP, who constitute a significant proportion of the MDD group. Additionally, our study provides different biological data from the immunological and neurotrophins data with the advantages of well controlled pharmacological treatment and closely longitudinal follow-up, which might reduce possible confounders^26^. BDNF and the cytokines that we measured in the present study have been proposed as biomarkers for BP^18^,^31^,^43^. Our findings reinforce the necessity to explore the role of these potential biomarkers for treatment responses, disease activity, and prognosis of SBP.

Our study has some limitations. This is the first study to investigate the pharmacological effect of typical BP treatment for SBP patients, but our study population was not large enough to allow us to draw definite conclusions. Additional studies with larger samples are required to confirm our preliminary findings. Our study had strict limitations for concomitant medications and was well controlled for many possible confounding factors. However, we did not explore other factors, such as disease course, smoking, BMI and other metabolic parameters, which might have affected the correlation between proinflammatory factors. We investigated only the anti-inflammatory cytokine TGF-β1. Future studies that measure other anti-inflammatory cytokines in SBP patients are needed. Moreover, the dropout rate in the current study was around 40%. Although our dropout rate was not much higher than those in most BP clinical trials^59^, we hypothesized that some of the dropouts had less efficacious responses to treatment, which might have biased our interpretation of our results. Additionally, we only included patients with shorter- duration hypomania in current study, which constituted only part of subthreshold bipolar patients. Generalizing our results to all subthreshold patients will require additional data. The lack of a control group of SBP patients treated with antidepressants only is another limitation. However, the possible risk of antidepressant-induced mania or hypomania and cycle acceleration in SBP patients remains a major concern. Therefore, our findings should be interpreted with caution.

## Conclusion

The present longitudinal study shows the treatment response similarities of SBP and BP-II patients, and supports a broader definition for hypomania. In addition, the changes in BDNF and some cytokine levels in SBP patients were different from those in BP-II patients. Our findings support the need to increase the duration of follow-ups of clinical severity, prognosis, and the role of these potential biomarkers in patients with SBP in future studies.

## Additional Information

**How to cite this article**: Wang, T.-Y. *et al*. Comparing clinical responses and the biomarkers of BDNF and cytokines between subthreshold bipolar disorder and bipolar II disorder. *Sci. Rep.* **6**, 27431; doi: 10.1038/srep27431 (2016).

## Figures and Tables

**Table 1:** Demographic and clinical data, BDNF and cytokine levels in the SBP and BP-II groups.

	**Baseline**			**Endpoint**		
	**SBP (n = 41)**	**BP-II (n = 48)**	**P**	**SBP (n = 22)**	**BP-II (n = 30)**	**P**
Age(years)	28.5 ± 10.2	31.5 ± 11.3	0.19	27.8 ± 9.2	32.8 ± 10.6	0.08
Sex (Male/Female)	21/20	26/22	0.78	10/12	20/10	0.13
Disease duration(years)	11.9 ± 10.1	13.8 ± 10.5	0.43	10.3 ± 9.3	15.7 ± 10.7	0.08
Plasma level of VPA (μg/mL)	NA	NA		69.1 ± 22.7	69.5 ± 20.1	0.96
Concomitant medication						
Risperidone(mg/day)	0.49 ± 0.58	0.44 ± 0.62	0.69	0.59 ± 0.80	0.35 ± 0.59	0.22
Fluoxetine(mg/day)	7.32 ± 6.72	7.08 ± 5.82	0.86	9.09 ± 6.84	7.67 ± 6.26	0.44
Lorazepam(mg/day)	1.18 ± 0.75	1.42 ± 1.11	0.24	1.43 ± 1.36	1.53 ± 1.18	0.78
Group psychotherapy (With/Without)	9/32	11/37	0.91	5/17	9/21	0.56
HDRS	18.9 ± 5.1	19.5 ± 4.7	0.61	8.6 ± 4.4	9.6 ± 4.9	0.43
YMRS	9.3 ± 4.4	8.3 ± 4.1	0.27	4.6 ± 1.7	4.6 ± 2.9	0.93
TNF-α(pg/ml)	1.78 ± 1.37	1.49 ± 1.48	0.35	1.28 ± 1.18	1.30 ± 1.43	0.95
TGF-β1(pg/ml)	34309.58 ± 21683.80	38714.49 ± 17693.52	0.20	31827.78 ± 17675.51	39418.76 ± 20249.08	0.17
IL-8(pg/ml)	2.81 ± 3.91	2.49 ± 2.21	0.63	2.20 ± 3.62	2.62 ± 2.83	0.64
IL-6(pg/ml)	1.05 ± 0.80	1.92 ± 2.24	0.02*	1.08 ± 1.57	1.43 ± 2.42	0.56
IL-1β(pg/ml)	1.62 ± 1.96	0.81 ± 0.64	0.03*	0.89 ± 0.63	1.21 ± 1.84	0.51
BDNF(pg/ml)	14913.63 ± 7592.23	19208.40 ± 9927.70	0.03*	14856.85 ± 7189.91	21200.85 ± 11136.25	0.02*

Data are mean ± standard deviation, unless otherwise indicated. NA: Not available.

^*^P < 0.05.

**Table 2:** Comparisons of treatment response, cytokines and BDNF levels between patients with SBP and BP-II during 12 weeks of pharmacological treatment.

	**SBP**		
	**Estimate**	**t**	**P**
Model 1			
HDRS	−0.19	−0.25	0.81
YMRS	0.14	0.35	0.73
Model 2			
BDNF	−4452.70	−2.92	0.005*
TNF-α	0.41	1.63	0.11
TGF-β1	−6970.26	−2.34	0.02*
IL-6	−0.80	−1.93	0.06
IL-8	0.30	0.57	0.57
IL-1β	0.60	2.00	0.05

^*^P < 0.05.

Reference group: BP-II group. Model 1: Covarying for age, gender, treatment duration, disease duration, concomitant group psychotherapy and medications. Model 2: Covarying for age, gender, treatment duration, disease duration, concomitant group psychotherapy and medications, HDRS and YMRS scores.
